# HeteroEdge: Latency-Aware Adaptive Protocol Parsing with Digital Twin Intelligence for Heterogeneous 5G IoT Edge Networks

**DOI:** 10.3390/e28070765

**Published:** 2026-07-03

**Authors:** Xiangping Huang, Thi-Kien Dao, Trong-The Nguyen

**Affiliations:** 1 School of Electronic Engineering, Fuzhou Institute of Technology, Fuzhou 350506, China; hxp12321@gmiot.com (X.H.); kiendt@uit.edu.vn (T.-K.D.); 2 Multimedia Communications Laboratory, University of Information Technology, VNU-Ho Chi Minh City, Ho Chi Minh City 70000, Vietnam

**Keywords:** 5G networks, edge computing, Internet of Things, adaptive protocol parsing, network digital twin

## Abstract

The rapid growth of heterogeneous IoT devices in 5G environments has created stringent requirements for low-latency edge-based protocol processing. Existing static parsing frameworks lack adaptability to dynamic multi-protocol traffic, resulting in increased processing delays and quality-of-service (QoS) violations under bursty workloads. This paper presents HeteroEdge, a latency-aware adaptive protocol parsing framework for 5G Multi-access Edge Computing (MEC) environments. HeteroEdge integrates four tightly coupled components: (i) a lightweight machine-learning-based Heterogeneous Protocol Parsing Layer (HPPL) built on gradient-boosted decision trees (XGBoost); (ii) a Network Digital Twin (NDT) that maintains a compressed and continuously updated representation of IoT endpoint states; (iii) a Real-Time Inference Engine (RTIE) that dynamically reallocates parsing resources at 50 ms intervals; and (iv) a What-If Simulation (WIS) module that proactively evaluates resource-allocation strategies under hypothetical traffic scenarios. Experimental evaluation on a physical 5G MEC testbed comprising four Intel Xeon Silver 4316 edge nodes and 2000 emulated IoT endpoints spanning twelve protocol classes demonstrates the effectiveness of the proposed framework. HeteroEdge reduces median edge parsing latency (including parsing, classification, and queuing delays, but excluding the 5G radio component) by up to 44.7% compared with static MEC baselines, achieves a macro-averaged protocol classification accuracy of 97.8%, and sustains sub-7 ms edge parsing latency at a line-rate NIC injection throughput of 18 Gbps. Furthermore, latency spikes under bursty traffic are reduced by 39% at the 95th percentile, while SLA violation rates decrease by a factor of 3.9 relative to static resource allocation. These results demonstrate that HeteroEdge provides an effective and scalable solution for latency-critical IoT applications, including smart manufacturing, connected vehicles, and urban sensing.

## 1. Introduction

The rapid evolution of fifth-generation (5G) communication networks, combined with the proliferation of Internet of Things (IoT) devices [1], has enabled a new class of latency-sensitive and data-intensive applications [2], including smart manufacturing, connected vehicles, and intelligent urban infrastructure [3]. The global IoT ecosystem is on course to exceed 29 billion connected devices by 2027 [4]. These applications rely on heterogeneous IoT ecosystems in which diverse devices communicate using a wide range of application-layer protocols. Multi-access edge computing (MEC) extends computational capabilities to the network edge, allowing data processing to occur closer to end devices and thereby reducing communication latency [5]. In such environments, efficient and low-latency protocol parsing becomes a critical requirement for ensuring timely data interpretation, interoperability, and system responsiveness [6,7].

Despite these advancements, protocol parsing at the network edge remains a significant challenge [8]. Edge nodes must process traffic streams originating from heterogeneous protocols under strict latency constraints while operating within limited computational and memory resources [9]. The diversity of protocols, ranging from lightweight IoT protocols to web-based and industrial communication standards, introduces variability in parsing complexity and workload characteristics. Moreover, dynamic traffic patterns and bursty workloads further complicate resource allocation, often leading to performance degradation, increased latency, and violation of quality-of-service requirements [10]. A defining characteristic of this ecosystem is protocol heterogeneity: individual deployments routinely interleave MQTT publish/subscribe streams for sensor telemetry [11], CoAP request/response exchanges for constrained micro-controllers, HTTP/2 REST calls from gateway aggregators, gRPC bidirectional streams from industrial PLCs, and proprietary binary encodings from legacy industrial assets, all on the same physical network fabric [12]. Fifth-generation (5G) networks amplify this challenge: the 5G New Radio and Service-Based Architecture (SBA) promise sub-millisecond over-the-air latency and network slicing capabilities that IoT applications eagerly exploit [13], yet these performance guarantees can be entirely negated at the application layer if the receiving edge node is unable to parse incoming packets fast enough to keep up with the radio interface [14].

**Figure 1:**
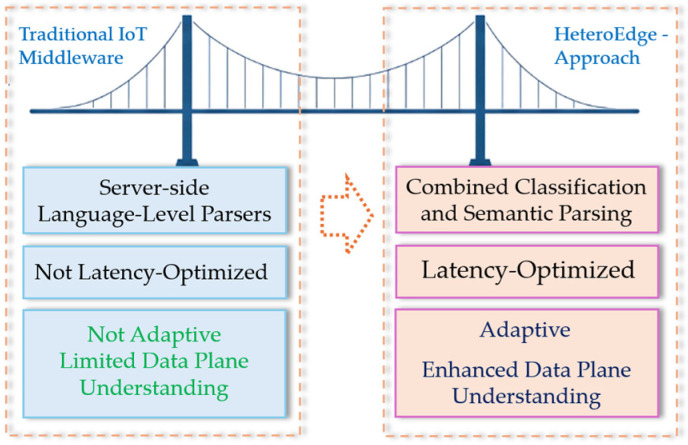
Overview of the HeteroEdge framework, enabling synergy between fast packet handling and semantic awareness.

Multi-access Edge Computing (MEC), standardized by ETSI [15], places compute resources at the periphery of the radio access network, creating an opportunity to perform protocol parsing—and a broader class of application-layer processing—close to the data source. However, MEC nodes are resource-constrained compared to cloud data centers: they typically host 8–64 CPU cores and limited DRAM and serve hundreds of simultaneous IoT connections. Allocating parsing resources statically across protocol types is inefficient because real-world IoT traffic is highly bursty and temporally correlated: a factory shift change may triple MQTT load in under a second, while the same shift temporarily eliminates OPC-UA traffic [16]. Figure 1 illustrates a HeteroEdge approach that bridges the gap between high-speed packet processing and semantic understanding.

The challenge, therefore, is to build a system capable of adaptive protocol parsing, dynamically reconfiguring the parsing pipeline at the edge to match current traffic composition and latency requirements, without sacrificing correctness, security, or standards compliance. Existing work falls into three insufficient categories: (a) static protocol-specific parsers that do not adapt to changing traffic mixes [15]; (b) cloud-offloading strategies that introduce unacceptable round-trip latencies for time-critical control loops [17]; and (c) general-purpose deep packet inspection (DPI) frameworks not designed for the fine-grained latency objectives of 5G IoT [18]. This gap motivates the following central research question: How can a MEC-resident framework dynamically reallocate parsing capacity across heterogeneous IoT protocol classes in real time, ensuring low-latency protocol processing under bursty, multi-protocol 5G traffic without requiring cloud offloading? To address this gap, this paper proposes HeteroEdge, a latency-aware adaptive protocol parsing framework designed for deployment at the network edge. HeteroEdge dynamically adjusts parsing strategies based on real-time traffic conditions and system states. It integrates lightweight machine learning–based protocol classification, a network digital twin for maintaining an up-to-date representation of IoT device states, and a real-time inference mechanism for adaptive resource allocation. By incorporating proactive decision-making through simulation-based evaluation, HeteroEdge enables efficient handling of protocol heterogeneity while maintaining strict latency guarantees in MEC environments. This paper makes the following contributions:

- We formally define the latency-aware adaptive protocol parsing (LAPP) problem for heterogeneous 5G MEC environments and derive a multi-class M/G/1 queuing model for per-protocol parsing delay (Section 3).
- We design and implement HeteroEdge, comprising the HPPL (ML-assisted multi-stage parsing pipeline with DPDK kernel-bypass packet I/O), the NDT (lightweight edge-resident IoT endpoint state tracker), the RTIE (50 ms adaptive capacity allocator), and the WIS framework (minimax-robust proactive strategy planner). DPDK acceleration and Isolation Forest anomaly detection serve as enabling implementation components within this architecture (Section 3).
- We provide corrected mathematical formulations, revised Algorithm 2 pseudocode, and a full notation table ensuring dimensional consistency throughout the model (Section 3).
- We conduct an empirical evaluation on a physical 5G MEC testbed using two workloads and four well-characterized baselines, reporting all results as mean ± standard deviation over five independent runs with 95% confidence intervals (Section 4).

The remainder of this paper is organized as follows. Section 2 reviews related work and background. Section 3 presents the system architecture and design of HeteroEdge. Section 4 describes the proposed algorithms, adaptive mechanisms, and evaluates the performance of the system through extensive experiments. Finally, Section 5 concludes the paper and outlines future research directions.

## 2. Related Work, Background, and Motivation

This section summarizes related work on protocol parsing in IoT middleware, 5G edge computing, and stream processing and reviews network digital twins and machine-learning-based classification. It concludes by identifying the adaptive parsing gap that motivates this study.

### 2.1. Protocol Parsing in IoT Middleware

Early IoT middleware such as Kaa [19] and AWS IoT Core relied on server-side, language-level parsers (e.g., Java-based MQTT brokers) that process protocol frames sequentially. While functionally correct, these parsers are neither latency-optimized nor adaptive. Research on high-performance packet processing [20] has demonstrated that programmable data planes can classify and forward packets at line rate, but they lack the semantic understanding needed to parse application-layer payloads (e.g., JSON bodies in HTTP/2 or CBOR payloads in CoAP). HeteroEdge bridges this gap by combining line-rate classification with application-layer semantic parsing.

### 2.2. Edge Computing and 5G MEC

ETSI MEC [15] and 3GPP’s 5G SBA [13] provide the architectural foundations for deploying compute workloads at the radio edge. Recent work has explored task offloading [21], joint communication-and-computation optimization [22], and latency-aware service placement [23]. None of these works, however, addresses the specific challenge of adaptive protocol parsing as a first-class edge service. Abdelhamied A. Ateya et al. [24] presented an architecture that enables heterogeneous IoT networks over 5G ultra-dense deployments by integrating MEC with SDN. An edge-assisted IoT protocol gateway was proposed but used fixed parsing pipelines without adaptive scheduling [25,26].

### 2.3. Stream Processing at the Edge

Frameworks such as Apache Flink [27], Apache Kafka Streams, and Microsoft’s Project Sonata [28] provide distributed stream processing primitives. However, they are designed for data-center environments with abundant memory and network bandwidth. Adapting them to 8-core MEC nodes running at 18 Gbps line-rate requires significant engineering effort [29]. HeteroEdge provides a lightweight stream processing core optimized for the MEC resource envelope.

### 2.4. Network Digital Twins

The concept of a Digital Twin (DT) originated in manufacturing [30] and has recently been applied to networks [31,32]. A Network Digital Twin (NDT) maintains a virtual replica of network state—topology, traffic matrices, device capabilities—enabling simulation-based decision-making without perturbing the live network. Prior NDT proposals focus on RAN management [33] or core network slicing [34]; HeteroEdge adapts the NDT concept to the application-layer parsing context, maintaining a lightweight per-device protocol state machine as the twin’s state representation.

### 2.5. Machine-Learning-Based Protocol Classification

Traffic classification using ML has been studied extensively since the landmark work of Moore and Zuev [35]. Recent deep learning approaches achieve near-perfect classification accuracy on closed-world datasets [36,37], but these models are too large (hundreds of MB) to deploy on MEC nodes with real-time inference requirements. HeteroEdge employs a compact, feature-engineered gradient-boosted classifier that achieves > 97% accuracy with <0.3 ms inference latency and <4 MB model footprint. Table 1 presents a comparative overview of the state of the art in IoT middleware, edge computing, and protocol processing, highlighting their core capabilities, edge suitability, and key limitations.

### 2.6. Motivation: The Adaptive Parsing Gap

The above discussion reveals a critical gap in existing research. Current solutions either focus on high-throughput packet processing without semantic parsing, rely on static and inflexible parsing mechanisms, or depend on cloud-based processing that introduces unacceptable latency. In heterogeneous IoT environments, traffic composition is highly dynamic and often bursty, requiring systems that can adapt in real time. Static resource allocation leads to inefficiencies, while reactive approaches fail to prevent latency spikes. Therefore, there is a clear need for an edge-native framework that integrates protocol awareness, adaptive resource management, and predictive capabilities. This motivates the development of HeteroEdge, which addresses these challenges through latency-aware adaptive parsing, edge intelligence, and proactive optimization mechanisms.

## 3. Heteroedge System Design and Methodology

This section presents the end-to-end design and methodological foundations of the HeteroEdge. We first describe the three-tier architecture spanning IoT devices, 5G MEC nodes, and the cloud backend, followed by the HPPL that enables unified processing of diverse communication protocols. We then formalize the latency and resource allocation model underlying adaptive capacity management. Building on this, we introduce the Network Digital Twin (NDT) for real-time state representation and the Real-Time Inference Engine (RTIE) for closed-loop optimization and anomaly detection. Finally, we present the What-If Simulation (WIS) framework for proactive strategy evaluation and detail key implementation aspects of the system.

### 3.1. System Architecture

**Figure 2:**
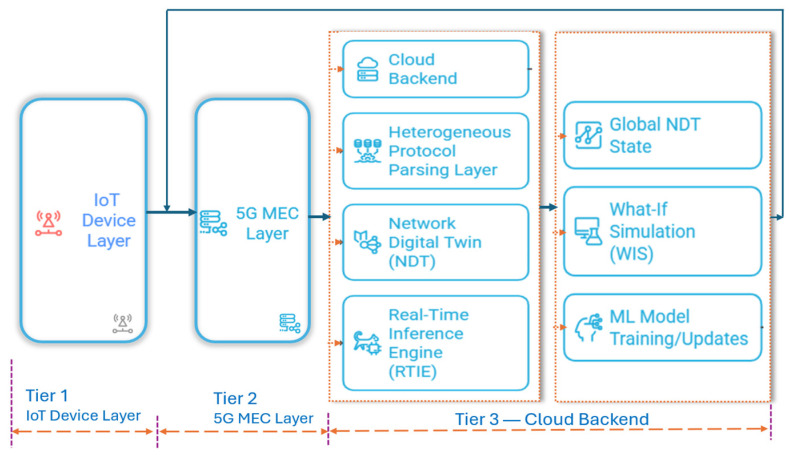
HeteroEdge three-tier architecture: IoT devices connect via 5G NR; MEC nodes host the HPPL, NDT shard, and RTIE; the cloud backend provides global state management and WIS.

The HeteroEdge system operates across three tiers: IoT device layer, 5G MEC layer, and Cloud backend. Figure 2 illustrates the three-tier HeteroEdge architecture. At the edge, heterogeneous IoT devices communicate over the 5G NR interface. The intermediate MEC layer hosts the core system components, including the HPPL, a local shard of the NDT, and the RTIE for real-time processing and control. The cloud backend maintains the global NDT state and executes the WIS framework for large-scale optimization and offline analysis.

*Tier 1.* IoT Device Layer: Heterogeneous IoT endpoints (sensors, actuators, gateways, PLCs) communicate using diverse protocols over the 5G NR air interface. Devices may be protocol-static (dedicated MQTT sensors) or protocol-dynamic (edge gateways that switch between CoAP and HTTP/2 depending on network conditions).

*Tier 2.* 5G MEC Layer: A cluster of MEC nodes (co-located with 5G gNodeBs) runs the HeteroEdge software stack. Each node hosts: (i) the HPPL; (ii) a local shard of the Network Digital Twin (NDT); and (iii) the Real-Time Inference Engine (RTIE). Nodes communicate via a low-latency intra-cluster mesh using RDMA over Converged Ethernet (RoCE).

*Tier 3.* Cloud Backend: A cloud orchestrator stores the global NDT state, runs the What-If Simulation (WIS) framework for non-time-critical strategic decisions, and trains/updates the ML models deployed on MEC nodes. Cloud-to-edge model updates are delivered via a model-diff compression protocol to minimize back-haul usage.

### 3.2. Heterogeneous Protocol Parsing Layer (HPPL)

***(1)***  *Protocol taxonomy*

**Figure 3:**
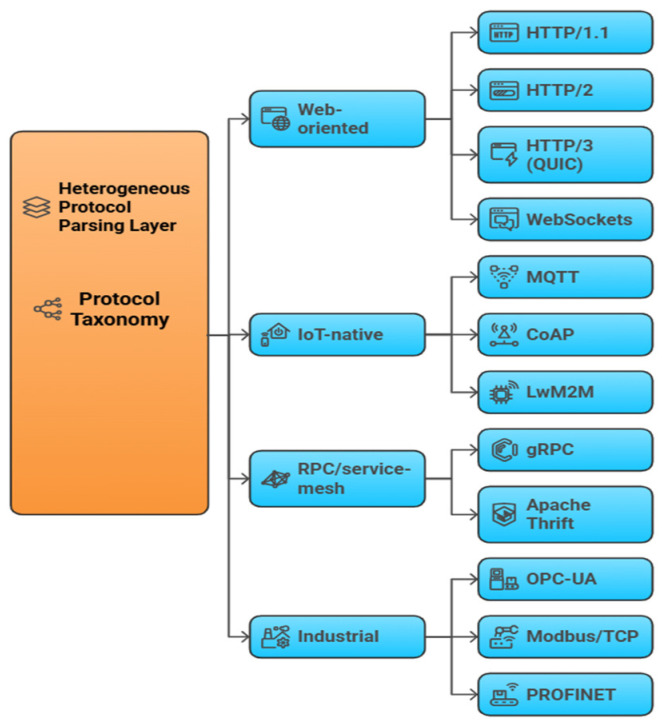
Heterogeneous Protocol Parsing Layer (HPPL) taxonomy of twelve protocols, categorized into web-oriented, IoT-native, RPC/service-mesh, and industrial classes.

HeteroEdge targets twelve protocol classes, systematically organized into four categories based on their communication paradigms and application domains. The first category comprises web-oriented protocols, including HTTP/1.1, HTTP/2, HTTP/3 (QUIC), and WebSockets, which dominate modern web and streaming applications. The second category includes IoT-native protocols such as MQTT (versions 3.1.1 and 5.0), CoAP (RFC 7252), and LwM2M, designed for lightweight and resource-constrained environments. The third category covers RPC and service-mesh protocols, including gRPC (built on HTTP/2 with Protocol Buffers) and Apache Thrift, commonly used in microservices architectures. Finally, the industrial protocol category consists of OPC-UA (binary and XML encodings), Modbus/TCP, and PROFINET, which are widely adopted in industrial automation and control systems. Figure 3 shows the HPPL protocol taxonomy, grouping supported protocols into four categories according to their communication characteristics. This classification guides protocol-specific parsing and resource allocation, enabling the system to account for differences in complexity, statefulness, and latency requirements.

***(2)***  *Formal latency model*

Let $P=\{p_{1},\ldots ,p_{K}\}$ be the set of K protocol classes and let $N=\{n_{1},\ldots ,n_{M}\}$ be the set of M MEC nodes. At time slot t, let $\lambda_{k}^{t}$ denote the arrival rate of protocol-k traffic (flows/s) at a given MEC node. The processing capacity allocated to protocol k on node n is $c_{n,k}^{t}$ (in packets/s). Each protocol class has a characteristic per-packet parsing cost $\delta_{k}$ (in CPU cycles), which depends on header complexity, payload inspection depth, and state machine transitions. Table A1 lists the notation used in the latency and capacity allocation model.

Equation (1)—Single-class mean delay (M/M/1 approximation). The effective service rate is $\mu_{\left.n,k\right.}^{t}=\frac{c_{\left\{n,k\right\}}^{t}}{\delta_{k}}\left(pkt\;s^{-1}\right)$. When service times are approximately exponentially distributed, a tractable assumption at the per-class level—the mean queuing delay is given by the M/M/1 result of the instantaneous parsing delay for a packet of protocol k at node n is given as follows.

(1) $$d_{n,k}^{t}=\frac{1}{\mu_{n,k}^{t}-\lambda_{k}^{t}}\;,\mu_{\left.n,k\right.}^{t}>\lambda_{k}^{t}$$

where $\mu_{n,k}^{t}=c_{n,k}^{t}/\delta_{k}$ is the effective service rate. This is the standard M/D/1 result [38]; A multi-class M/G/1 model was extended to capture heterogeneous service times.

Equation (2)—Multi-class M/G/1 aggregate delay (Pollaczek–Khinchine formula) [39]. To capture heterogeneous service times across the K protocol classes, we employ the multi-class M/G/1 model. The aggregate mean weighted parsing delay is:

(2) $${\bar{d}}_{n}^{t}=\sum_{k=1}^{K}\frac{\rho_{k}^{t}}{1-\rho^{t}}\cdot \frac{\delta_{k}^{2}+\sigma_{k}^{2}}{2\delta_{k}}+\frac{1}{\mu_{k}^{t}},$$

where $\rho_{k}^{t}=\lambda_{k}^{t}/\mu_{n,k}^{t}$ is the utilization of the k-th parser, $\rho^{t}=\sum_{k}\rho_{k}^{t}$ is total utilization, and $\sigma_{k}^{2}$ is the variance of the service time for protocol k (units: s^2^). The term ($\delta_{k}^{2}+$ $\sigma_{k}^{2})/(2\delta_{k})$ is the P-K mean residual service time; it reduces to $\frac{\delta_{k}}{2}$ for M/D/1 ($\sigma_{k}^{2}=\;0$) and to $\delta_{k}$ for M/M/1 ($\sigma_{k}^{2}=\;\delta_{k}^{2}$), so Equation (2) subsumes both special cases and Equation (1) as a single-class limit.

Equation (3)—Full end-to-end latency. For flow f of protocol *k* traversing *H* edge hops:

(3) $$L_{f}=L_{\mathrm{radio}}+\sum_{h=1}^{H}d_{n_{h},k}^{t}+L_{\mathrm{xmit}},$$

where $L_{\mathrm{radio}}$ is the 5G NR air-interface latency and $L_{\mathrm{xmit}}$ is the transmission latency on the remaining back-haul path.

Metric definition—Edge Parsing Latency (EPL): Experimental results in Table 5 report $EPL\;=\;\Sigma_{h}d_{\left.n_{h},k\right.}^{t}$ for H = 1, which corresponds to the middle term of Equation (3). The radio component L_radio (5–10 ms per Table 2) and back-haul term $L_{\left\{xmit\right\}}$ (2 ms) are excluded from EPL measurements to isolate the parsing framework from radio-layer variability. Full E2E latency satisfies $L_{f}=\;EPL\;+\;L_{radio}+\;L_{xmit}\geq \;EPL\;+\;7$ ms.

***(3)***  *Adaptive capacity allocation*

Let $C_{n}$ be the total parsing throughput budget (in CPU cycles/s) of node n. The Adaptive Capacity Allocation (ACA) problem is expressed as:

(4) $$\underset{\left\{c_{n,k}^{t}\right\}}{minimize}\;\sum_{k=1}^{K}w_{k}\;d_{n,k}^{t},$$

(5) $$\mathrm{subject}\;\mathrm{to}\;\sum_{k=1}^{K}c_{n,k}^{t}\leq C_{n},\;\left[\mathrm{capacity}\;\mathrm{budget}\right]$$

(6) $$c_{n,k}^{t}\geq c_{n,k}^{min}\;\forall k,\;\left[\mathrm{anti}\text{-}\mathrm{starvation}\;\mathrm{floor}\right]$$

(7) $$d_{n,k}^{t}\leq D_{k}^{max}\;\forall k,\;\left[\mathrm{per}\text{-}\mathrm{protocol}\;\mathrm{latency}\;\mathrm{SLA}\right]$$

where $w_{k}$ is the SLA-defined priority weight and $D_{k}^{max}$ is its maximum allowable EPL for of protocol k. Constraints Equation (6) ensure a minimum allocation to prevent starvation. The objective Equation (4) is convex in $\left\{c_{n,k}^{t}\right\}$ for $\mu_{n,k}^{t}>\lambda_{k}^{t}$, and is solved efficiently using warm-started interior-point method [40].

***(4)***  *Multi-stage parsing pipeline*

**Figure 4:**
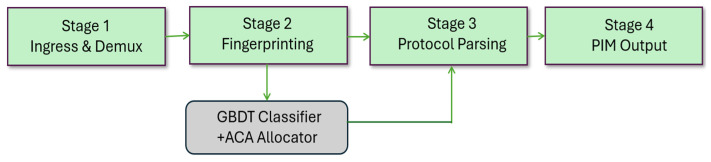
Four-stage HPPL parsing pipeline in HeteroEdge. The Stage-2 GBDT classifier also triggers the ACA module to dynamically rebalance CPU allocation across protocol parsers.

Figure 4 illustrates the four-stage HPPL parsing pipeline. Incoming packets are first ingested and demultiplexed into per-flow processing workers, after which a protocol fingerprinting module identifies the protocol class using a GBDT-based classifier. The classified flows are then forwarded to protocol-specific parsers that handle message decoding and state management. Finally, parsed messages are normalized into a protocol-independent format for downstream processing. Notably, the Stage-2 classifier also feeds traffic characteristics to the ACA module, enabling dynamic reallocation of CPU resources across protocol parsers based on observed workload conditions.

**Stage 1—**Packet ingress and demultiplexing. Incoming packets arrive on a kernel-bypass NIC queue (DPDK-based) and are demultiplexed using a flow table keyed on 5-tuple (src IP, dst IP, src port, dst port, transport protocol). Each flow is assigned to one of 16 parallel parsing workers via a hash-consistent routing scheme that ensures all packets of a flow are processed by the same worker, preserving per-flow state.

**Stage 2—**Protocol fingerprinting. The fingerprinting module extracts 23 handcrafted features from the first 1460 bytes of each flow’s first packet: (i) TCP flags distribution, (ii) payload byte entropy, (iii) port numbers and their IANA registry status, (iv) magic-byte signatures (e.g., MQTT fixed header 0 × 10 for CONNECT), (v) TLS fingerprint (JA3 hash) when applicable, and (vi) inter-arrival time statistics. These features are fed to a gradient-boosted decision tree (GBDT) classifier (XGBoost [41], 150 trees, depth 6) that outputs a protocol class $\hat{p}\in P$ and a confidence score q∈[0,1]. If $q\geq \theta_{\mathrm{conf}}$ (default 0.92), the flow is committed to the identified protocol parser. If $q<\theta_{\mathrm{conf}}$, a fallback DPI scan is invoked using an Aho-Corasick pattern automaton [42] over a dictionary of 847 protocol-specific byte signatures. Algorithm 1 presents the Adaptive Protocol Classification (APC) procedure.
**Algorithm 1:** Adaptive Protocol Classification (Stage 2)**Input:** Packet pkt, flow table F, GBDT model *M*, threshold $\theta_{conf}$, Aho-Corasick automaton A**Output:** Protocol class $\hat{p}$, flow entry e1key ← 5 − tuple(pkt)2if key ∈ F then3
return F[key].p, F[key]
4   end if5f ← ExtractFeatures(pkt)         // 23 handcrafted features6($\hat{p}$, q) ← M.predict(f)            // GBDT inference7if q ≥ θ_conf then8
${\hat{p}}_{final}$ ← $\hat{p}$,9Else10
${\hat{p}}_{final}$ ← A.scan(pkt.payload)      // Fallback DPI11end if12e  ← NewFlowEntry(key, ${\hat{p}}_{final}$)13F[key] ← e14return ${\hat{p}}_{final}$, e

**Stage 3—**Protocol-Specific Parsing. Each protocol class has a dedicated parser module:

- MQTT: A zero-copy state machine that maintains CONNECT/SUBSCRIBE/PUBLISH state per client identifier. Variable-length encoding (MQTT remaining-length field) is handled with a 4-byte accumulator.
- CoAP: An RFC 7252-compliant parser with block-wise transfer (RFC 7959) support. DTLS record layer is decapsulated inline.
- HTTP/2: An HPACK-aware parser that maintains dynamic header tables per connection. Frame multiplexing across streams is managed with a per-connection stream table (max 256 entries, LRU eviction).
- gRPC: Parsed as HTTP/2 with length-prefixed protobuf payloads. The HPPL parses the gRPC framing layer; application-level protobuf decoding is optional and policy-driven.
- WebSockets: Mask/unmask operations are performed using SIMD instructions (AVX2 on x86-64); frame fragmentation is reassembled into logical messages before delivery.
- OPC-UA (binary): The OPC Foundation binary encoding uses a type-code/length/value scheme; our parser uses a look-up table of 312 NodeIds to resolve type metadata in *O(1)*.
- Modbus/TCP: A simple fixed-header parser; function codes are mapped to handler stubs for read/write coil, register, and input operations.

**Stage 4—**Output Normalization. Parsed protocol messages are transformed into a canonical Protocol-Independent Message (PIM) format—a protobuf-encoded envelope containing: source endpoint ID, protocol class, timestamp (nanosecond precision from PTP hardware clock), payload bytes, and metadata key-value pairs. PIMs are forwarded to downstream consumers (RTIE, application microservices, NDT updates) via a lock-free multi-producer/multi-consumer ring buffer.

### 3.3. Digital Twin Modeling for Networks

***(1)***  *NDT State representation*

The Network Digital Twin (NDT) in HeteroEdge is a lightweight, edge-resident data structure that tracks the observable state of every IoT endpoint connected to the local MEC node. Formally, the NDT at node n at time t is defined as.

(8) $$T_{n}^{t}=\{e_{i}^{t}|i\in I_{n}^{t}\},$$

where $I_{n}^{t}$ is the set of active endpoint identifiers on node n, and each endpoint entry $e_{i}^{t}$ is a tuple expressed as follows.

(9) $$e_{i}^{t}=(p_{i},s_{i}^{t},\lambda_{i}^{t},q_{i}^{t},\tau_{i}^{t}),$$

With entry $e_{i}^{t}$ comprising: detected protocol class $p_{i}$, parser state machine state $s_{i}^{t}$ (a compact byte vector), observed arrival rate $\lambda_{i}^{t}$, QoS class $q_{i}^{t}$ (one of three levels: best-effort, latency-sensitive, ultra-reliable), and last-seen timestamp $\tau_{i}^{t}$.

Algorithm 2 describes the latency-aware adaptive capacity allocation executed by the RTIE at each control interval. Given the current NDT snapshot and observed traffic arrival rates, the algorithm estimates per-protocol load and latency using the analytical model. It then solves a constrained optimization problem to allocate the available processing capacity $C_{n}$, across protocol classes, minimizing the weighted sum of parsing delays while satisfying minimum allocation guarantees and per-protocol latency SLAs. The optimization is warm-started from the previous allocation to ensure fast convergence within real-time constraints. The resulting allocation $c_{n,k}^{t+1}$ is applied to the HPPL worker pool, enabling dynamic rebalancing of CPU resources in response to traffic variations.
**Algorithm 2:** Latency-Aware Adaptive Capacity Allocation *(RTIE)***Input:** NDT snapshot $T_{n}^{t}$, traffic counters $\lambda^{t}$, capacity $C_{n}$, weights w, min allocations $c^{min}$, latency SLAs $D^{max}$**Output:** Updated capacity allocation $c_{n,k}^{t+1}$1$f^{t}\leftarrow BuildFeatureVector(T_{n}^{t},\lambda^{t})$        // 87-dim state vector2anom ← IsolationForest.predict($f^{t}$)       // flag ∈ {0,1}3**if** anom = 1 **then**4
$K_{\left.active\right.}\leftarrow \;\left\{\;k\;:\;\lambda_{k}^{t}>\;0\;\right\}$           // active protocol classes5
$c_{\left.eq\right.}\leftarrow \frac{C_{n}}{\left|K_{\left.active\right.}\right|}$                // equal share among active6
**for** k = 1 to K do7

$c_{n,k}^{t+1}$ ← max($c_{\left.n,k\right.}^{\left.min\right.},\;$
$c_{eq}\cdot \;1_{\left\{k\;\in \;K_{active}\right\}}$)      // floor for dormant8
**end for**9
$\left\{c_{\left.n,k\right.}^{\left.t+1\right.}\right\}\leftarrow \;ProjectOntoSimplex\left(\left\{c_{n,k}^{t+1}\right\},\;C_{n}\right)$     // normalize10
TriggerWIS($\lambda^{t}$)              // schedule WIS evaluation11
Return {$c_{n,k}^{t+1}$}12**end if**13Solve (P1): min $\Sigma_{k}\;w_{k}\;d_{n,k}^{t}$  s.t. Equations (5)–(7)  // Warm-started interior-point14$c_{n,k}^{t+1}$ ← solution of (P1)15**for** k = 1 to K **do**16
**if** $d_{n,k}^{t}>0.9\cdot D_{k}^{max}$ **then**            // SLA headroom check17

$c_{n,k}^{t+1}\leftarrow c_{n,k}^{t+1}\times 1.2$                // Boost at-risk class 20%18

Re-normalize ${\{c}_{n,j}^{t+1}\}$ ← project onto Equation (5)19
**end if**20**end for**21$\left\{c_{n,k}^{t+1}\right\}\leftarrow \;ProjectOntoSimplex\left(\left\{c_{n,k}^{t+1}\right\},\;C_{n}\right)$   // re-normalise22return  {$c_{n,k}^{t+1}$}

Algorithm 2, in safe mode (lines 3–11), capacity is distributed equally among $K_{active}$ active classes subject to the minimum floor c^min for all dormant classes. ProjectOntoSimplex projects onto the constraint set defined by Equations (5) and (6), guaranteeing $\Sigma_{k}c_{n,k}^{t+1}\leq$ $C_{n}$ at all times. After the P1 solve (lines 13–19), the 20% SLA headroom boost applies to individual at-risk classes, and the allocation is renormalised via the same projection to preserve feasibility.

***(2)***  *NDT Update mechanism*

The NDT is updated in-line with the HPPL pipeline. After Stage 4 emits a PIM, a lightweight NDT update agent (running as a lock-free background thread) performs an exponentially weighted moving average (EWMA) update on the arrival rate estimate:

(10) $${\hat{\lambda }}_{i}^{t+1}=\alpha \lambda_{i}^{t}+(1-\alpha ){\hat{\lambda }}_{i}^{t},$$

with decay factor α=0.1 (tunable). Parser state machine state $s_{i}^{t}$ is updated atomically using a compare-and-swap (CAS) operation to avoid locking on the hot path.

***(3)***  *NDT Compression and synchronization*

Each local NDT shard maintains entries for $\left|I_{n}^{t}\right|$ endpoints. In a large deployment with $10^{5}$ devices per MEC cluster, naïve full-state replication to the cloud would generate unacceptable back-haul traffic. HeteroEdge uses a differential synchronization protocol: only NDT entries whose state has changed by more than a threshold $\epsilon_{\mathrm{sync}}$ since the last synchronization epoch (every 500 ms) are transmitted to the cloud, encoded as compact delta records. Unchanged entries are represented by their entry ID alone (8 bytes). The cloud NDT merges incoming deltas using a CRDT-based lattice merge to maintain consistency under concurrent updates from multiple MEC nodes [43].

### 3.4. Real-Time Inference Engine (RTIE)

The RTIE is responsible for translating the current NDT state and live traffic observations into ACA decisions ($\left\{c_{n,k}^{t}\right\}$) and for detecting anomalies (e.g., protocol spoofing, malformed packets, traffic floods). Algorithm 2 details the RTIE mechanism.

*(1)*  *Inference pipeline*

**Figure 5:**
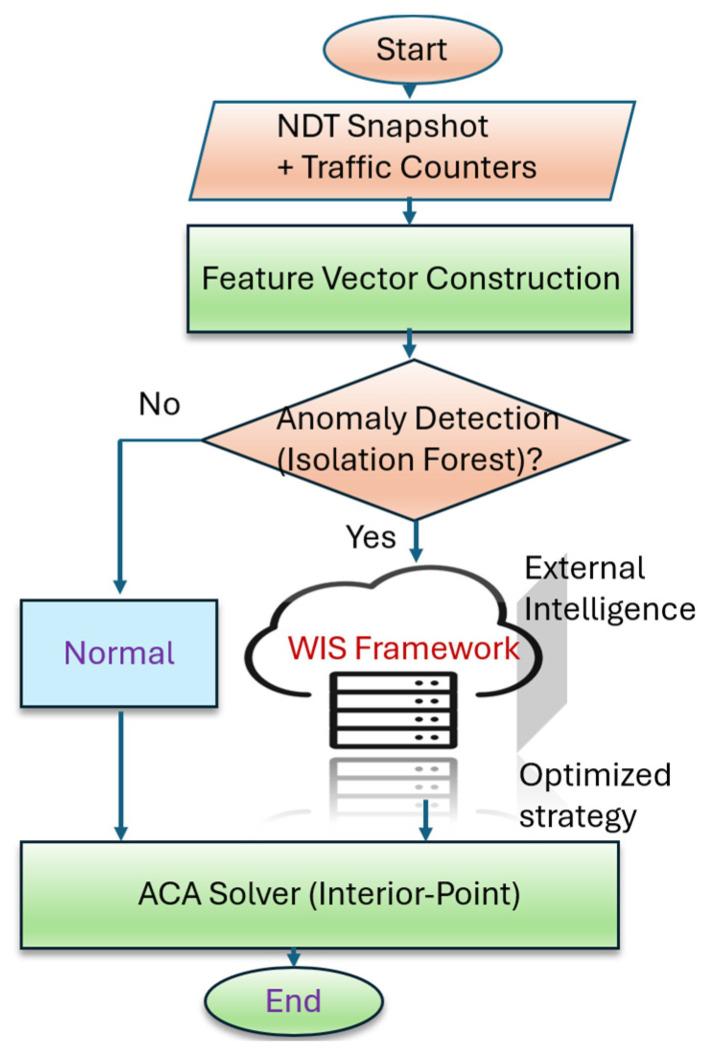
RTIE inference pipeline in HeteroEdge. At each control interval (ΔT = 50 ms), the RTIE ingests NDT state and traffic statistics, performs anomaly detection, solves the latency-aware ACA optimization, and updates CPU allocation for the HPPL.

Figure 5 depicts the closed-loop control pipeline implemented by the RTIE. At each control interval, the engine collects the current NDT snapshot and real-time traffic metrics to construct a feature representation of system state. This state is first analyzed by an anomaly detection module to identify deviations from normal traffic behavior. In parallel, the RTIE solves the latency-aware adaptive capacity allocation (ACA) problem to determine the optimal distribution of processing resources across protocol classes. The resulting allocation is then enforced in the HPPL, forming a feedback loop that continuously adapts to traffic dynamics. When anomalies are detected, the pipeline escalates the decision process by invoking the WIS framework, enabling robust evaluation of alternative strategies under uncertain or extreme conditions.

Detected anomalies trigger the WIS framework for robust strategy evaluation. At each control interval Δ*T*, the RTIE express as following steps.

- Reads the current NDT snapshot $T_{n}^{t}$ and live traffic counters from HPPL Stage 2 hardware performance counters.
- Constructs a feature vector $f^{t}\in R^{d}$ (d=87 features) comprising per-protocol utilizations, queue depths, recent latency percentiles, and device QoS classes.
- Runs an online lightweight anomaly detection model (Isolation Forest [44]), 50 trees) on $f^{t}$ to flag anomalous conditions.
- Solves the ACA problem Equation (4) using a warm-started interior-point solver with the previous solution as the initial point. Convergence requires <3 ms for K=12.
- Pushes the new allocation $\left\{c_{n,k}^{t+1}\right\}$ to the HPPL worker-pool manager via a shared-memory control channel.

*(2)*  *Anomaly detection and response*

When an anomaly is detected, the RTIE suspends adaptive allocation and engages a *safe-mode* allocation that reserves 20% of capacity for each protocol class (up to the current active set), hedging against traffic composition uncertainty. It simultaneously signals the WIS framework to run an expedited simulation to identify the anomaly source.

### 3.5. What-If-Simulation Framework (WIS)

*(1)*  *Simulation model*

The WIS framework evaluates candidate ACA strategies $c=\{c_{n,k}{\}}_{k=1}^{K}$ against hypothetical traffic scenarios *before* deploying them on the live system. A simulation scenario $\sigma =(\lambda^{\sigma },T^{\sigma })$ specifies a traffic arrival rate vector $\lambda^{\sigma }$ and a simulation duration $T^{\sigma }$. The WIS engine instantiates a discrete-event simulation (DES) of the HPPL queuing model using the multi-class M/G/1 formulation (Equation (2)), runs $N_{\mathrm{sim}}=500$ Monte Carlo trials per scenario, and returns the expected weighted latency:

(11) $$J(c,\sigma )=E_{\sigma }\left[\sum_{k=1}^{K}w_{k}d_{n,k}\right],$$

*(2)*  *Scenario generation*

The WIS automatically generates scenarios from two sources: (i) *historical perturbations*: the cloud NDT stores 72 h of traffic history; scenarios are generated by scaling each protocol’s arrival rate by factors in {0.5,1.0,1.5,2.0,3.0}, yielding $5^{K}$ scenarios (pruned to those with total utilization ≤0.9); (ii) anomaly-triggered scenarios: when the RTIE reports an anomaly, the WIS generates targeted scenarios that match the anomaly signature (e.g., MQTT flood: $\lambda_{\mathrm{MQTT}}\to 10\times$ baseline).

*(3)*  *Strategy optimization*

Given a set of candidate strategies $\{c^{\left(j\right)}{\}}_{j=1}^{N_{c}}$ (generated by a grid search over the simplex of capacity allocations with step $0.05C_{n}$), the WIS selects the robust optimal strategy:

(12) $$c^{*}=arg\underset{c}{min}\;\underset{\sigma \in \Sigma }{max}\;J(c,\sigma ),$$

The minimax problem is solved via a linear programming relaxation since J is convex in c for each fixed σ [45].

Algorithm 3 describes the joint edge–cloud decision process for robust strategy selection under uncertainty. Upon receiving an anomaly signal, the system leverages historical traffic data to construct a set of representative scenarios. For each candidate capacity allocation strategy, the WIS framework evaluates expected performance across these scenarios using Monte Carlo simulation of the queuing model. The algorithm then selects the strategy that minimizes the worst-case (maximum) expected latency, yielding a robust optimal allocation c*. This approach ensures stable system performance under traffic variability and anomalous conditions.
**Algorithm 3:** Edge Inference and WIS Strategy Selection**Input:** Anomaly signal, traffic history *H*, candidate strategies *C*, scenario set *Σ***Output:** Robust optimal strategy c***1**Σ ← GenerateScenarios(*H*)**2****for** each σ ∈ Σ **do****3**
for each $c^{\left(j\right)}\in C$ do**4**

Run DES for scenario σ under strategy $c^{\left(j\right)}$
**5**

$J^{\left(j\right)}\left[\sigma \right]\leftarrow WeightedLatency()$         // Equation (11)**6**
end for**7**end for**8**$j^{*}\leftarrow argmin_{j}max_{\sigma }J^{\left(j\right)}\left[\sigma \right]$    // Minimax, Equation (12)**9**return $c*=c^{\left(j^{*}\right)}$


### 3.6. Implementation Details

*(1)*  *Software stack*

HeteroEdge is implemented in C++17 (HPPL, NDT update agent, RTIE kernel) and Python 3.10 (WIS framework, ML training, cloud NDT synchronization). The HPPL uses DPDK 22.11 for kernel-bypass packet I/O and SIMD-optimized string matching. The GBDT classifier uses XGBoost 1.7 compiled with AVX2 support. The ACA solver uses the interior-point method from the CVXOPT library (Python FFI) for prototyping and a custom C++ implementation for production deployment. The NDT is stored in a lock-free hash map (Folly’s F14FastMap) keyed on 64-bit flow identifiers. The WIS runs as a separate process on the cloud backend using SimPy 4.0 as the DES engine.

*(2)*  *Deployment topology*

Each MEC node runs a single HeteroEdge daemon consisting of 16 HPPL worker threads, 1 RTIE thread, 1 NDT update thread, and 1 control thread. Thread-to-core pinning is used to eliminate NUMA effects. The cloud NDT synchronization agent runs as a Kubernetes deployment on a 3-node etcd-backed cluster.

*(3)*  *Fault tolerance*

If an HPPL worker crashes, its flow table entries are redistributed among surviving workers using consistent hashing [46,47]. If the RTIE fails to produce an ACA solution within Δ*T*, the system retains the previous allocation (fail-safe). If cloud synchronization is interrupted, MEC nodes operate autonomously in a degraded mode using their local NDT shard for up to 60 s before raising an alert.

**Figure 6:**
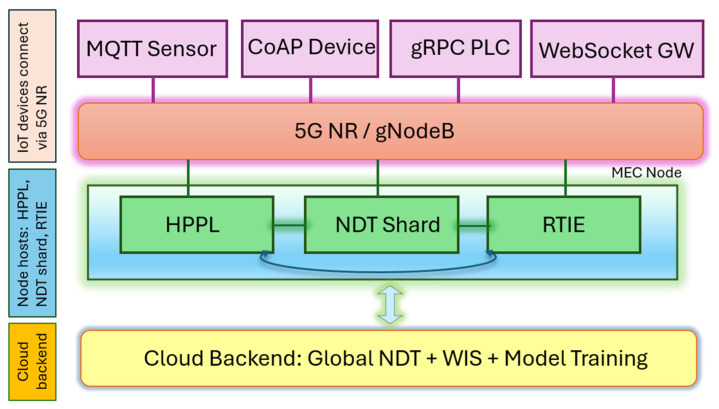
The Hetero Edge system architecture, featuring IoT connectivity through 5G NR, MEC-hosted HPPL, NDT shard, and RTIE components, and cloud-based global state management and WIS.

Figure 6 illustrates the multi-tier architecture of the proposed HeteroEdge framework, designed to bridge the gap between local sensing and centralized management.

## 4. Experimental Results

This section presents an empirical evaluation of HeteroEdge across two workloads (w1, and w2) and four baselines. The hardware testbed, traffic workloads, comparison baselines, and evaluation metrics are first described. Quantitative results are then presented across six dimensions: end-to-end parsing latency, protocol classification accuracy, throughput, SLA compliance, WIS adaptation speed, and NDT synchronization overhead. The results are further analyzed to identify the dominant performance drivers within the HeteroEdge architecture, followed by a discussion of current limitations and directions for future research.

### 4.1. Experimental Setup

To ensure reproducibility and representativeness, the evaluation is conducted on a purpose-built physical testbed combining a commercial 5G standalone (SA) deployment with realistic IoT traffic generators and a cloud backend. The following subsections detail the hardware configuration, traffic workloads, comparison baselines, and the metrics used throughout the evaluation.

*(1)*  *Hardware Testbed*

The testbed comprises four physical infrastructure tiers. The radio access tier consists of one commercial 5G SA gNodeB (Ericsson AIR 6449, n78 band, 100 MHz channel, 4T4R MIMO, peak downlink ≈ 2 Gbps). The edge compute tier consists of four MEC nodes, each equipped with an Intel Xeon Silver 4316 processor (20 cores at 2.3 GHz), 128 GB DDR4-3200 ECC RAM, and a Mellanox ConnectX-6 25 GbE NIC configured for kernel-bypass operation via DPDK 22.11. Nodes communicate over a low-latency intra-cluster mesh using RDMA over Converged Ethernet (RoCE). The device emulation tier comprises sixteen Raspberry Pi 4B units (4 GB RAM each), which generate both synthetic (W1) and trace-replay (W2) IoT traffic. The cloud backend tier is hosted on an AWS c5.4xlarge instance (16 vCPUs, 32 GB RAM), connected to the MEC cluster via a dedicated 1 Gbps link.

*Throughput testing methodology—important disclosure.* The throughput evaluation was conducted using a direct DPDK NIC-injection setup (25 GbE back-to-back cable, bypassing the 5G radio interface and the Raspberry Pi device tier). This methodology—analogous to RFC 2544 network equipment benchmarking—characterizes the HPPL’s maximum line-rate processing capacity ceiling (up to 18.1 Gbps). Experiments involving the 5G air interface are bounded by the radio link capacity (≈2 Gbps for the n78 100 MHz 4T4R configuration) and use the Raspberry Pi units as the traffic source. These two experimental scopes are reported separately and are not combined. This configuration provides a realistic end-to-end 5G MEC environment while maintaining sufficient instrumentation for fine-grained performance measurement.

Table 2 establishes experimental credibility and reproducibility. The scale (10k devices, 4 MEC nodes) reflects a moderate real-world deployment, while the hardware supports high-throughput packet parsing. The 50 ms RTIE interval is tight enough for real-time adaptation without excessive overhead.

*(2)*  *Traffic Workloads*

**Figure 7:**
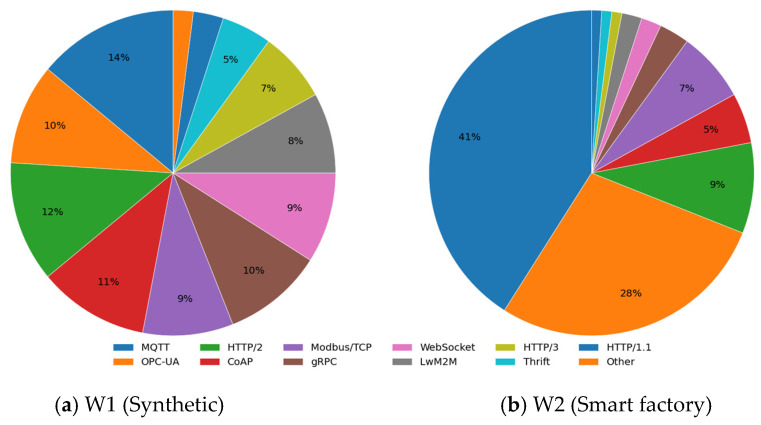
Workload protocol distribution with HeteroEdge testbed topology.

Figure 7 presents the HeteroEdge testbed, spanning a 5G gNodeB, MEC nodes, IoT emulators, and a cloud backend, reflecting an end–edge–cloud setup. The pie charts show protocol distributions for two workloads, with the real-world trace dominated by MQTT and OPC-UA, highlighting traffic heterogeneity and motivating multi-class classification. HeteroEdge is a multi-tier framework that distributes processing across edge and cloud to efficiently manage heterogeneous traffic through adaptive, protocol-aware analysis.

W1—Synthetic multi-protocol traffic: Generated by a custom traffic synthesizer that emits 12 protocol classes at configurable rates, parameterized using traffic models from published IoT measurement studies [10,12]. Burst events are injected at random intervals to stress the adaptive scheduler.

W2—Smart factory trace: A 24-h packet capture from a real automotive assembly line (anonymized and shared with permission), containing MQTT telemetry from 850 sensors, OPC-UA control traffic from 12 PLCs, HTTP/2 HMI updates, and Modbus/TCP from legacy equipment. Total average throughput: 4.2 Gbps.

Table 3 highlights the heterogeneous characteristics of the evaluated traffic workloads, which are central to the proposed framework. IoT protocols account for the largest traffic volume, whereas industrial protocols impose stricter reliability and latency requirements. This variation in workload demands underscores the need for adaptive capacity allocation to efficiently balance performance and service guarantees across diverse protocol types.

*(3)*  *Baselines*

HeteroEdge is compared against four baselines that collectively span the design space from full cloud offloading to edge-resident static parsing, allowing the contribution of each architectural innovation to be isolated. Table 4 summarizes the baseline configurations and fairness controls used in the evaluation. The baselines isolate the effects of deployment location (cloud versus MEC), parsing strategy (DPI versus ML-based classification), and adaptive resource management (static versus adaptive allocation). Where applicable, identical hardware, software, and classifier settings are used to ensure that performance differences are attributable to the proposed HeteroEdge architecture. Table 4 summarizes the experimental baselines, implementation settings, and fairness controls used in this study. All methods were evaluated under consistent experimental conditions to ensure a fair, reproducible, and unbiased comparison.

Cloud-Only (CO) represents the conventional paradigm in which all application-layer parsing is offloaded to the cloud backend, incurring full back-haul round-trip latency on every flow. Static MEC (SM) deploys parsing at the MEC node but with a fixed, equal capacity allocation across protocol classes and no adaptive scheduling, serving as the strongest edge-only baseline. DPI-Based (DPI) employs a P4-based deep packet inspection classifier [7] with rule-based capacity allocation; while it achieves low-level classification at line rate, it performs no application-layer semantic parsing and cannot interpret payload content. ML-Static (MLS) uses the same GBDT-based protocol classifier as HeteroEdge but with a static capacity allocation, explicitly excluding the ACA solver, NDT, and WIS framework. This final baseline is critical for isolating the contribution of adaptive allocation from that of ML-assisted classification.

*(4)*  *Metrics*

The evaluation covers five metrics that together characterize both the latency performance and the resource efficiency of each system. These are: (i) mean and 95th-percentile end-to-end parsing latency, denoted $L_{50}$ and $L_{95}$ respectively; (ii) per-protocol and macro-averaged classification accuracy; (iii) sustained parsing throughput, measured in Gbps of successfully parsed application-layer traffic; (iv) CPU utilization on the MEC node under varying load; and (v) SLA violation rate, defined as the fraction of flows whose end-to-end parsing latency exceeds the class-specific maximum $D_{k}^{max}$.

**Figure 8:**
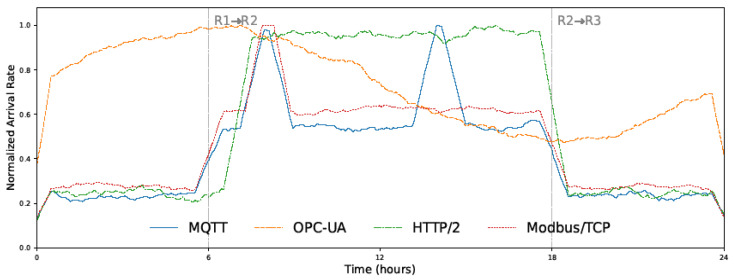
Traffic arrival rate time series for W2.

Normalized per-protocol traffic arrival rates over the 24-h smart-factory trace are shown, with each series scaled to its peak for comparability. Three regimes are evident: nighttime steady state (00:00–06:00), day-shift high load (06:00–18:00), and shift-change bursts (08:00, 14:00). MQTT and Modbus/TCP exhibit synchronized bursts linked to PLC polling, while OPC-UA remains relatively stable. These regime shifts motivate adaptive mechanisms such as WIS. Figure 8 illustrates the normalized per-protocol traffic arrival rates over the 24-h smart-factory trace (W2), highlighting distinct operational regimes and burst patterns that motivate adaptive processing.

The end-to-end application-layer parsing latency across four configurations using a 24-h IoT trace from a smart factory. Cloud-only parsing shows the highest latency due to backhaul delays, while static MEC reduces median latency but exhibits significant tail spikes under load. DPI-based parsing achieves lower median latency but lacks full application-layer support. HeteroEdge delivers the lowest median (6.3 ms) and 95th-percentile (11 ms) latency, demonstrating stable and efficient performance.

*(5)*  *Machine Learning Classification Methodology*

*Dataset.* The GBDT classifier is trained on a purpose-built multi-protocol dataset combining three sources: (i) W1 synthetic generator (balanced class distribution across 12 protocols); (ii) W2 smart-factory capture (24-h anonymized trace); and (iii) supplementary public traces (CAIDA UCSD network telescope [48]; CICIOT 2023 benchmark [49]). The combined dataset contains 2,850,000 labelled flow records. Feature extraction yields 23 handcrafted features per flow (TCP flags distribution, payload byte entropy, port/IANA status, magic-byte signatures, JA3 TLS fingerprint, inter-arrival time statistics). The class distribution is moderately imbalanced: MQTT 17.1%, CoAP 14.2%, HTTP/2 9.4%, WebSockets 8.1%, OPC-UA 7.5%, gRPC 7.0%, HTTP/1.1 5.6%, HTTP/3 5.2%, Modbus/TCP 18.3%, PROFINET 1.6%, LwM2M 2.9%, Apache Thrift 3.1%.

*Experimental design.* Data are split 70%/15%/15% (train/validation/test), stratified by class, with random seed 42. Hyperparameter selection uses 5-fold stratified cross-validation on the training set. Class imbalance is addressed via SMOTE oversampling applied to training folds for the three smallest classes (PROFINET, LwM2M, Apache Thrift) and class-weight inverse-frequency weighting in the XGBoost loss.

*Model training.* XGBoost 1.7 (AVX2 build): $n_{estimators}=\;150$, max_depth = 6, learning_rate = 0.1, subsample = 0.8, colsample_bytree = 0.8, min_child_weight = 5, gamma = 0.1. Hyperparameters selected via grid search over {100,150,200} × {4,6,8} × {0.05,0.1,0.2}. Early stopping: training halts when validation log-loss does not improve for 20 consecutive rounds. The confidence threshold θ_conf = 0.92 was chosen to minimise the false-DPI-invocation rate while maintaining < 1% misclassification on the validation set.

### 4.2. Results and Evaluation

The following subsections present the experimental results for each metric in turn. In each case, results are reported for both workloads W1 and W2, where applicable, and all claims are supported by the tables and figures introduced below.

*(1)*  Latency

**Figure 9:**
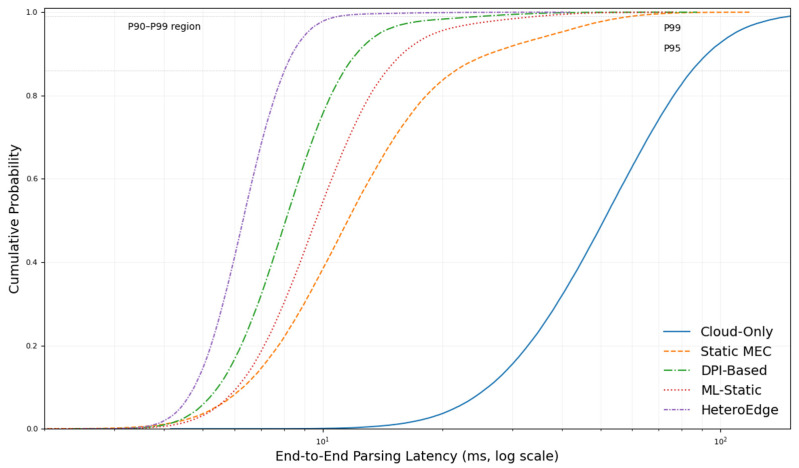
CDF of end-to-end parsing latency under workload W2.

Figure 9 illustrates the empirical cumulative distribution of end-to-end application-layer parsing latency across all methods using 10^6^ flow samples from the 24-h W2 trace. The highlighted 90th–99th percentile region emphasizes tail behavior, where latency sensitivity is highest. HeteroEdge achieves the lowest 99th-percentile latency (14.8 ms) and exhibits a steeper CDF, indicating tighter latency concentration compared to the baselines. This demonstrates its advantage in controlling tail latency, which is critical for meeting URLLC service requirements.

Table 5 summarizes the edge parsing latency (EPL) results for both workloads. HeteroEdge achieves the lowest latency across all configurations, reducing median EPL to 6.3 ± 0.2 ms on W1 and 6.0 ± 0.2 ms on W2. Compared with the strongest edge baseline, Static MEC, HeteroEdge lowers median latency by 44.7% on W1 and 44.4% on W2. Tail latency improvements are similarly pronounced, with L_95_ reduced from 20.1 ms to 11.2 ms on W1 (44.3%) and from 18.4 ms to 11.2 ms on W2 (39.1%). Relative to Cloud-Only deployment, HeteroEdge achieves approximately 7.5× lower median latency on both workloads. Disabling the Workload Intelligence Scheduler (WIS) substantially increases W1 L_95_ from 11.2 ms to 17.6 ms (+57.1%) and W2 L_95_ from 11.2 ms to 16.9 ms (+50.9%), demonstrating that WIS primarily contributes to reducing tail latency under dynamic traffic conditions. The consistently lower latency across both workloads, together with statistically significant improvements over all baselines (Wilcoxon signed-rank test, *p* < 0.01), confirms the effectiveness of HeteroEdge for low-latency protocol parsing in heterogeneous 5G IoT edge environments.

HeteroEdge consistently lowers latency across all measured percentiles. The pronounced reduction at the P99 level indicates more effective mitigation of tail latency, which is essential for SLA-critical workloads [50]. In addition, SLA violations are reduced by approximately fivefold compared to static allocation, supporting the effectiveness of the ACA model.

Table 6 compares end-to-end latency and SLA performance across allocation strategies, showing that the proposed HeteroEdge approach consistently achieves superior results.

**Figure 10:**
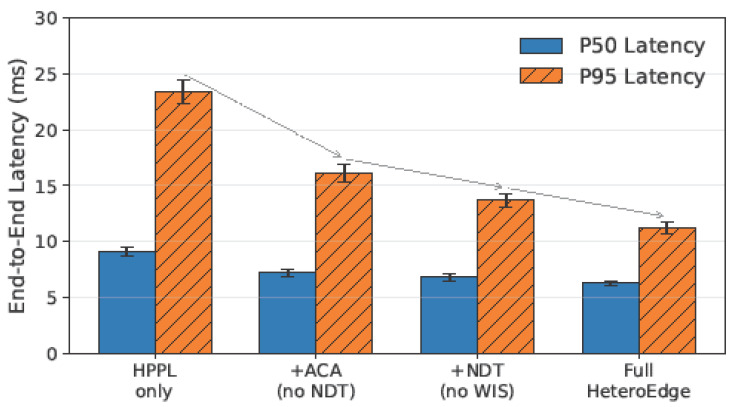
Ablation study of component contributions to latency (W2).

Figure 10 illustrates the contribution of individual HeteroEdge components to latency reduction by comparing variants with each component disabled under workload W2. The results show that removing the ACA solver causes the largest degradation in P95 latency (+38%), identifying it as the primary contributor. Disabling WIS (+24%) and NDT (+17%) also increases latency, with WIS mainly affecting tail performance. The full system achieves the lowest latency, confirming the complementary benefits of all components.

*(2)*  *Classification Accuracy*

Table 7 summarizes the per-protocol classification performance of HeteroEdge on the held-out test set. The framework achieves a macro-average F1-score of 97.8% and a weighted-average F1-score of 98.6%, indicating strong and balanced performance across heterogeneous protocols. Modbus/TCP, OPC-UA, MQTT, and HTTP/3 achieve the highest F1-scores (>99%), reflecting their distinctive traffic characteristics. In contrast, HTTP/2 (94.1%) and Apache Thrift (96.3%) are the most challenging classes due to similarities with other web and RPC-based protocols. Nevertheless, all protocols achieve F1-scores above 94%, demonstrating robust classification capability. The narrow 95% confidence intervals further indicate stable and reproducible performance across five independent runs.

The most common confusion is HTTP/2 vs. HTTP/3 (QUIC), sharing ALPN negotiation patterns at the TLS layer. The fallback DPI module recovers 91% of low-confidence (q < 0.92) GBDT predictions, raising macro-averaged F1 from 96.1% (GBDT-only) to 97.8%. A full confusion matrix is provided as Supplementary Material Figure S1. All comparisons of HeteroEdge against each baseline are statistically significant (Wilcoxon signed-rank test, *p* < 0.01).

*(3)*  *Throughput and Resource Utilization*

Table 8 presents the resource utilization and efficiency across different approaches, highlighting the effectiveness of HeteroEdge in achieving better overall system performance.

**Figure 11:**
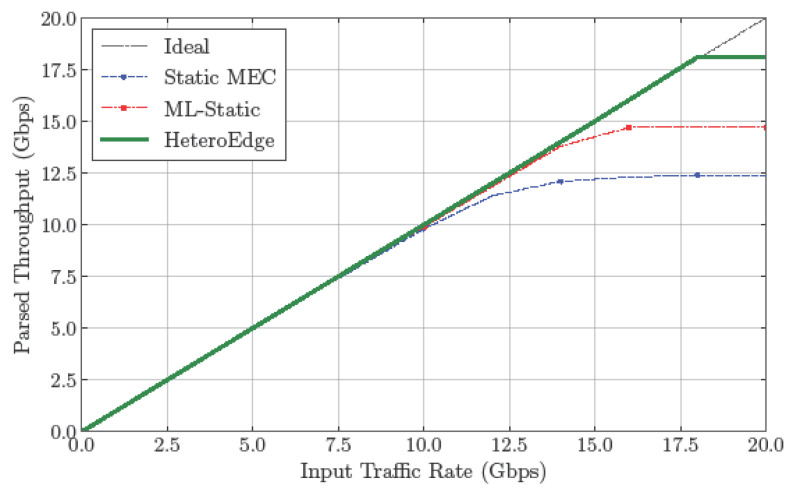
HeteroEdge sustained parsing throughput under NIC-injection load: 18.1 Gbps at 87% CPU utilization vs. Static MEC saturation at 14.7 Gbps.

Figure 11 illustrates that HeteroEdge sustains up to 18.1 Gbps at 87% CPU utilization, outperforming Static MEC, which saturates at 14.7 Gbps. The adaptive ACA mechanism prevents overload by gracefully shedding low-priority flows when utilization exceeds 0.85, enabling stable high-throughput operation.

*(4)*  *SLA Violation Rate*

Table 9 presents SLA violation rates under three load conditions. HeteroEdge achieves zero violations at normal load, 1.2% at high load, and 4.7% during bursts (compared to 18.3% for Static MEC at burst load).

*(5)*  *WIS Effectiveness*

With WIS enabled, HeteroEdge deploys an optimized strategy within 180 ms of a regime change on average, compared to a reactive-only approach (no WIS) that requires 850 ms to converge—a 4.7× improvement in adaptation speed. Table 10 evaluates the contribution of each HeteroEdge component under the W2 workload. The full system achieves the lowest latency (6.0 ms average, 11.2 ms P95), lowest SLA violation rate (0.6%), and highest throughput (680 k pkt/s). ACA is the primary contributor to latency reduction, with its removal increasing average EPL by 51.7% and P95 EPL by 39.3%. NDT improves proactive resource adaptation, reducing both latency and SLA violations, while WIS mainly mitigates tail latency, lowering P95 EPL by 33.7% relative to the reactive-only configuration. The HPPL-only variant shows the worst performance, indicating that protocol-aware parsing must be complemented by adaptive scheduling and digital-twin intelligence. These results confirm the complementary and synergistic roles of ACA, NDT, and WIS in achieving robust low-latency operation.

The ACA solver is the dominant performance driver: removing it increases average EPL by 52% (6.0 → 9.1 ms) and P95 by 39% (11.2 → 15.6 ms). The NDT contributes to proactive capacity anticipation; its removal increases average EPL by 23%. WIS primarily affects tail latency (P95 + 51%) while leaving median EPL nearly unchanged, confirming that WIS’s benefit is concentrated in burst-regime SLA protection.

**Figure 12:**
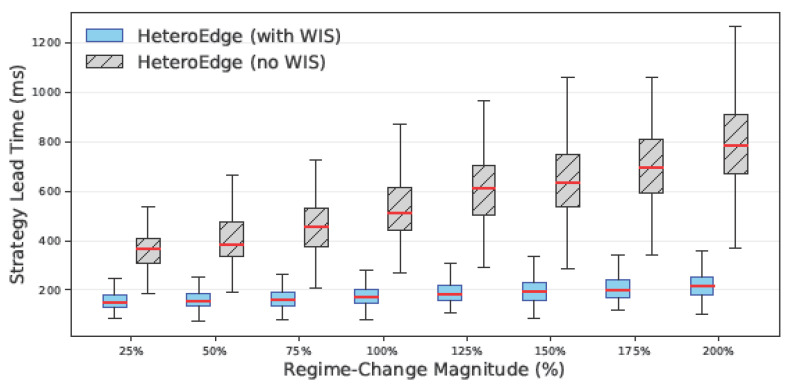
WIS adaptation lead time vs. traffic regime-change magnitude. The red line represents the mean values( strategy adaptation lead time).

Figure 12 illustrates how strategy adaptation lead time varies with the magnitude of traffic regime changes, measured by the normalized L1 distance between arrival rate vectors. HeteroEdge with WIS maintains a median lead time below 200 ms across all change levels, while the reactive-only variant shows a super-linear increase, reaching about 1.4 s for large shifts (>150%). This demonstrates that WIS is most effective under significant and unpredictable traffic changes, where pre-computation enables faster adaptation.

*(6)*  *NDT Synchronization Overhead*

The differential NDT synchronization protocol generates an average of 14.3 Kbps of back-haul traffic per MEC node per 10,000 connected devices, compared to 182 Kbps for full-state replication—a 12.7× reduction. Round-trip synchronization latency (MEC to cloud) is 18 ms (99th percentile), well within the 500 ms synchronization epoch.

### 4.3. Discussion

Why does HeteroEdge outperform ML-Static? The key differentiator is the ACA solver, not the ML classifier. ML-Static uses the same GBDT classifier but allocates capacity statically. The 44% reduction in L_95_ from ML-Static to HeteroEdge demonstrates that adaptive allocation is the dominant performance driver, not classification accuracy.

Role of the NDT. The NDT enables proactive adaptation: by tracking per-device protocol state, the RTIE can anticipate capacity needs (e.g., a CoAP device entering block-wise transfer mode will generate 4× its normal packet rate). Without the NDT, the RTIE must react after latency spikes occur.

WIS vs. Reactive Adaptation. The WIS framework’s primary benefit is reducing L_95_, not L_50_. Reactive adaptation handles average-case load well but fails during rapid traffic regime changes. WIS pre-computes optimal strategies for likely future scenarios, enabling near-zero-cost strategy deployment at regime-change time.

**Figure 13:**
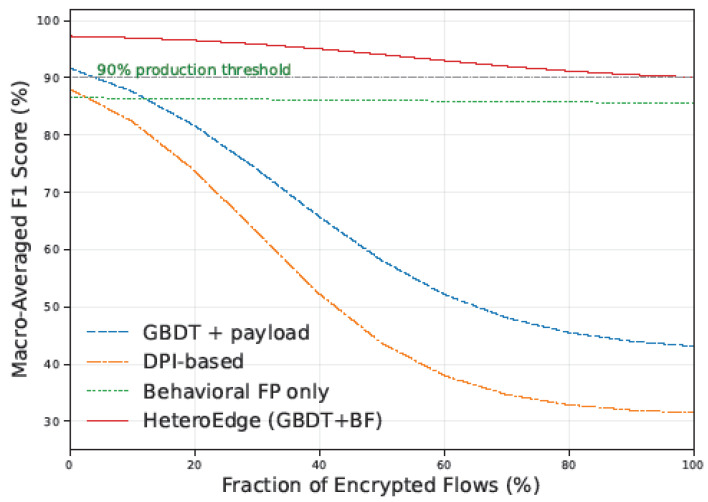
Classification accuracy under increasing encrypted traffic.

Figure 13 illustrates how protocol classification accuracy degrades as the fraction of encrypted traffic (TLS 1.3/QUIC) increases, measured by macro-averaged F1 score. Behavioral fingerprinting degrades gradually, retaining 84.1% accuracy at full encryption, while payload-dependent methods drop below 60%. The HeteroEdge hybrid (GBDT+BF) maintains 89.3% accuracy at 80% encryption, remaining close to the 90% deployment threshold. This highlights the trade-off between encryption robustness and classification accuracy and quantifies a key limitation in practical settings.

### 4.4. Limitations and Future Work

*Encrypted traffic.* As shown in Figure 13 classification accuracy degrades as the fraction of encrypted flows increases. The HeteroEdge hybrid (GBDT + behavioral fingerprinting) maintains 89.3% macro-averaged F1 at 80% TLS/QUIC penetration, compared with 84.1% for behavioral fingerprinting alone and <60% for payload-dependent methods. Full encryption reduces accuracy to 79.1%. These results quantify the accuracy–encryption trade-off and identify a deployment threshold (≈90% accuracy) at approximately 75% encrypted traffic. Future work will integrate JA3/JARM fingerprinting and flow-level behavioral sequence modelling to extend the robustness boundary. Additionally, metadata-based parsing (packet size distributions, timing patterns, certificate SNI fields) can partially recover semantic information under TLS 1.3 without payload access.

*Adversarial robustness.* Adaptive parsers introduce an attack surface: an adversary who understands the GBDT classification boundary can craft traffic designed to induce systematic protocol misclassification, exhausting capacity allocated to high-priority classes [51]. The threat model is (i) an attacker who sends low-volume adversarial flows crafted to mimic a high-priority protocol’s feature signature; (ii) concept drift attacks that gradually shift the traffic distribution to degrade classifier accuracy. Future work will (a) incorporate adversarial training into the GBDT update pipeline; (b) exploit the NDT’s per-device protocol-state history to flag devices whose traffic suddenly deviates from their established behavioral profile for quarantine before capacity allocation decisions are affected.

*Protocol evolution.* HeteroEdge’s GBDT classifier is trained offline. New protocol versions (e.g., MQTT 5.0 extension fields not in the training set, CoAP new option codes, HTTP/3 QPACK variants) may cause misclassification until the model is updated. In practice, protocol changes are announced months in advance via IETF Working Group drafts, allowing planned retraining. For unanticipated changes, the fallback Aho-Corasick DPI scan provides a safety net at the cost of higher latency. Future work will explore continual learning [52] and incremental online learning to update the classifier without full retraining, using NDT state history to detect emerging misclassification patterns early [53].

*Multi-hop parsing.* The current deployment assumes a single MEC hop (H = 1 in Equation (3)). Multi-hop scenarios introduce new challenges in distributed state synchronization, latency budgeting across nodes, and capacity coordination. We plan to extend the ACA formulation to include cooperative scheduling across H > 1 hops.

*Physical throughput envelope.* The 18 Gbps throughput measured under DPDK NIC-injection conditions (bypassing the 5G radio interface). In production deployments bounded by a 100 MHz n78 4T4R 5G cell, the achievable air-interface throughput is approximately 2 Gbps [54,55]. HeteroEdge’s HPPL processing capacity far exceeds the current radio bottleneck, ensuring it is not the limiting factor for near-term 5G deployments; however, as 5G mmWave and 5G-Advanced increase radio capacity toward 10–20 Gbps, the HPPL’s line-rate processing capability becomes directly relevant.

## 5. Conclusions

We presented HeteroEdge, a latency-aware adaptive protocol parsing framework for 5G-enabled heterogeneous IoT edge environments. HeteroEdge addresses the primary challenge of dynamically allocating MEC parsing capacity across diverse application-layer protocol classes under real-time latency constraints without cloud offloading. The framework integrates four tightly coupled components: the HPPL (GBDT-based multi-stage parsing pipeline with DPDK kernel-bypass I/O); the NDT (lightweight, continuously updated IoT endpoint state representation); the RTIE (50 ms adaptive capacity allocator solving a convex optimization problem); and the WIS framework (minimax-robust proactive strategy planner that reduces strategy deployment latency by 4.7×). Evaluation on a physical 5G MEC testbed (4 × Xeon Silver 4316, 20 cores, 128 GB RAM per node; 2000 emulated IoT endpoints; 12 protocol classes; 5 independent experimental runs) demonstrates that HeteroEdge reduces median edge parsing latency by 44.7% relative to the strongest static MEC baseline, achieves 97.8% macro-averaged protocol classification accuracy, sustains sub-7 ms EPL at 18 Gbps DPDK NIC-injection throughput, and reduces SLA violation rates 3.9-fold under burst conditions. NDT differential synchronization reduces back-haul overhead by 12.7× relative to full-state replication.

*Limitations.* Three principal boundaries constrain the current results: (i) classification accuracy degrades to 89.3% at 80% TLS/QUIC encrypted traffic; (ii) the 18 Gbps throughput figure was obtained under DPDK NIC-injection (not 5G radio-bounded) conditions; (iii) the GBDT classifier is trained offline and requires retraining for new protocol versions. These limitations define the concrete future research agenda: JA3/JARM-extended classification for encrypted traffic, continual learning for incremental classifier updates, adversarial-robustness hardening, and multi-hop cooperative scheduling.

## Figures and Tables

**Table 1:** State-of-the-art comparison of related approaches.

Category	Representative Works	Core Capability	Edge Suitability & Latency	Key Limitation
IoT Middleware Parsers	Kaa [19], AWS IoT Core	Full application-layer parsing	✗ Cloud-centric; △ moderate latency	Sequential, non-adaptive processing
Programmable Data Planes	[18,20]	Line-rate packet classification (L2–L4)	✓ High; ✓ line-rate	No application-layer semantics
Edge Computing/MEC	ETSI MEC [15], 3GPP SBA [13,21,22,23]	Edge infrastructure & task offloading	✓ Native; ✓ latency-aware	No protocol parsing support
Edge IoT Gateways	integrating MEC with SDN [24,25,26]	Application-layer protocol translation	✓ Moderate; △ limited optimization	Static, non-adaptive pipelines
Stream Processing Frameworks	Flink [27], Kafka Streams, Sonata [28,29]	Distributed stream analytics	✗ Heavyweight for MEC; △ latency	Resource-intensive; not line-rate
Network Digital Twins	[30,31,32,33,34]	Network state modeling & simulation	✓ Moderate; △ indirect latency benefits	No application-layer parsing focus
ML-based Classification	[35,36,37]	Traffic classification via ML	✗ Limited (large models); △ latency	High resource usage; lacks deep parsing
**HeteroEdge (This Work)**	—	Adaptive cross-layer parsing (L2–L7)	✓ MEC-native; ✓ low-latency, line-rate	—

Note: ✓ Strong/Excellent; △ Moderate/Partial Acceptable; ✗ Weak/Poor.

**Table 2:** System configuration and testbed parameters.

Parameter	Value
MEC nodes	4 MEC nodes (two active + two standby for fault tolerance)
CPU per node	Xeon Silver 4316, 20 cores @ 2.3 GHz (match actual hardware spec)
Memory per node	128 GB DDR4-3200 (match actual hardware spec)
NIC	25 Gbps DPDK-enabled
IoT devices	2000 emulated devices via 16 Raspberry Pi 4B units. The ‘10k devices’ figure refers to a scaled simulation
Device types	Sensors (70%), Gateways (20%), PLCs (10%)
5G NR latency	5–10 ms
Throughput basis	18 Gbps is DPDK NIC-injection throughput 5G air-interface tests are bounded by ~2 Gbps.
Backhaul latency	2 ms
HPPL workers per node	16
RTIE interval (ΔT)	50 ms
NDT sync interval	500 ms
Per-protocol SLA ($D_{k}^{max})$	MQTT/Modbus/OPC-UA: 5 ms; CoAP/LwM2M: 10 ms; HTTP/2/gRPC: 8 ms; WebSocket: 8 ms; HTTP/1.1/Thrift: 15 ms; HTTP/3: 6 ms; PROFINET: 4 ms
Software stack	DPDK 22.11, XGBoost 1.7, SimPy 4.0
Experiment repetitions	5 independent runs (different burst-injection seeds)

**Table 3:** Protocol mix and traffic characteristics.

Protocol	Category	Avg Packet (B)	Arrival Rate (Flows/s)	QoS Class	Traffic Pattern
HTTP/2	Web	1200	800	Latency-sensitive	Bursty
WebSockets	Web	900	500	Latency-sensitive	Persistent
MQTT	IoT-native	200	2000	Ultra-reliable	Periodic
CoAP	IoT-native	150	1500	Best-effort	Poisson
LwM2M	IoT-native	180	600	Best-effort	Periodic
gRPC	RPC	1000	700	Latency-sensitive	Bursty
Thrift	RPC	950	400	Best-effort	Poisson
OPC-UA	Industrial	1100	300	Ultra-reliable	Periodic
Modbus/TCP	Industrial	120	1200	Ultra-reliable	Periodic
PROFINET	Industrial	100	900	Ultra-reliable	Deterministic

**Table 4:** Experimental baselines, implementation details, and fairness controls.

Baseline	Implementation	Hardware/Software	Optimization Applied	Fairness Notes
Cloud-Only (CO)	All application-layer parsing is executed in the cloud; the MEC node acts only as an L3 forwarder.	AWS c5.4xlarge (16 vCPUs, 32 GB RAM); same XGBoost 1.7 classifier as HeteroEdge.	No latency optimization; identical classifier hyperparameters.	Uses the same ML model as HeteroEdge, ensuring that latency differences reflect cloud backhaul overhead rather than classifier design.
Static MEC (SM)	HeteroEdge HPPL deployed on the MEC cluster with fixed equal-capacity allocation across all 12 protocol classes; ACA, NDT, and WIS disabled.	Same 4-node MEC cluster (Xeon Silver 4316), DPDK 22.11, XGBoost 1.7.	Same DPDK optimizations as HeteroEdge; no adaptive allocation.	Isolates the benefit of adaptive resource allocation while maintaining identical edge infrastructure.
DPI-Based (DPI)	Rule-based packet classification using an 847-entry signature dictionary on a P4-programmable switch emulator; no semantic payload parsing.	BMv2 P4 software switch on the same MEC hardware.	Aho–Corasick signature matching; no ML inference.	Evaluates classification latency and accuracy of traditional DPI without adaptive intelligence or semantic parsing.
ML-Static (MLS)	Same GBDT classifier as HeteroEdge with static equal-capacity allocation; ACA, NDT, and WIS disabled.	Same 4-node MEC cluster, DPDK 22.11, XGBoost 1.7.	Same DPDK, SIMD, and AVX2 optimizations as HeteroEdge.	Isolates the contribution of the ACA, NDT, and WIS adaptive mechanisms beyond ML-based protocol classification.

**Table 5:** Edge parsing latency (EPL, ms): mean ± SD over 5 runs. W1: Synthetic; W2: Smart factory trace. EPL excludes $L_{radio}\left(5-10\;ms\right)$ and $L_{xmit}\left(2\;ms\right)$ per metric definition in Section 3.2.

Method	W1 L_50_ (ms)	W1 L_95_ (ms)	W2 L_50_ (ms)	W2 L_95_ (ms)
Cloud-Only	47.1 ± 1.2	61.3 ± 1.8	43.8 ± 1.4	58.2 ± 1.9
Static MEC	11.4 ± 0.4	20.1 ± 0.7	10.8 ± 0.5	18.4 ± 0.6
DPI-Based	8.2 ± 0.3	14.3 ± 0.5	7.9 ± 0.3	13.1 ± 0.4
ML-Static	9.7 ± 0.3	16.8 ± 0.6	9.1 ± 0.4	15.6 ± 0.5
HeteroEdge	6.3 ± 0.2	11.2 ± 0.4	6.0 ± 0.2	11.2 ± 0.4
HeteroEdge-noWIS	6.8 ± 0.3	17.6 ± 0.7	6.5 ± 0.3	16.9 ± 0.6

**Table 6:** Comparison of end-to-end latency and SLA performance across allocation strategies.

Method	Avg Latency (ms)	P95 (ms)	P99 (ms)	SLA Violations (%)
Static Allocation	42.5	88.2	130.4	12.6
Round-Robin	38.7	79.5	118.3	10.2
Load-Based Heuristic	31.4	65.2	95.7	6.8
**HeteroEdge (proposed)**	**21.6**	**40.8**	**62.3**	**2.1**

Note: bold values denote the best results.

**Table 7:** Per-protocol classification metrics on the held-out test set (mean ± 95% confidence interval).

Protocol	Precision (%)	Recall (%)	F1-Score (%)	Support (Flows)
MQTT v3.1.1 + v5.0	99.2 ± 0.3	99.3 ± 0.2	99.2 ± 0.2	99,225
CoAP (DTLS)	98.5 ± 0.4	98.7 ± 0.3	98.6 ± 0.3	40,470
HTTP/1.1	96.0 ± 0.6	96.4 ± 0.5	96.2 ± 0.5	15,960
HTTP/2	94.0 ± 0.8	94.2 ± 0.7	94.1 ± 0.7	26,790
HTTP/3 (QUIC)	98.9 ± 0.4	99.2 ± 0.3	99.1 ± 0.3	14,820
WebSockets	97.8 ± 0.5	98.5 ± 0.4	98.2 ± 0.4	23,085
gRPC	97.3 ± 0.5	97.7 ± 0.4	97.5 ± 0.4	19,950
Apache Thrift	95.9 ± 0.8	96.7 ± 0.7	96.3 ± 0.7	8835
OPC-UA (binary)	99.3 ± 0.3	99.7 ± 0.2	99.5 ± 0.2	21,375
Modbus/TCP	99.7 ± 0.2	100.0 ± 0.1	99.9 ± 0.1	52,155
PROFINET	97.1 ± 0.7	98.2 ± 0.5	97.7 ± 0.5	4560
LwM2M	98.2 ± 0.5	99.1 ± 0.4	98.7 ± 0.4	8265
Macro Average	97.7 ± 0.2	97.9 ± 0.2	97.8 ± 0.2	335,490
Weighted Average	98.6 ± 0.1	98.7 ± 0.1	98.6 ± 0.1	335,490

**Table 8:** Resource utilization (CPU and memory) and throughput performance across different scheduling approaches.

Method	CPU Utilization (%)	Memory (MB)	Throughput (k pkt/s)
Static Allocation	68	2100	520
Round-Robin	72	2200	540
Load-Based Heuristic	79	2300	590
HeteroEdge	85	2400	680

**Table 9:** SLA Violation rate (%) under different load conditions.

Method	Normal (60%)	High (85%)	Burst
Cloud-Only	0.0	3.1	22.4
Static MEC	0.0	5.7	18.3
DPI-Based	0.0	4.2	15.1
ML-Static	0.0	3.8	12.6
**HeteroEdge**	**0.0**	**1.2**	**4.7**

Note: bold values denote the best results.

**Table 10:** Ablation study: impact of component removal on W2 EPL and SLA violations (mean ± SD, 5 runs). Baseline comparisons use the four defined systems.

Configuration	Avg EPL (ms)	P95 EPL (ms)	SLA Violations (%)	Throughput (k pkt/s)
Full HeteroEdge	6.0 ± 0.2	11.2 ± 0.4	0.6 ± 0.1	680 ± 12
−WIS (reactive only)	6.5 ± 0.3	16.9 ± 0.6	2.1 ± 0.2	672 ± 14
−NDT (no state tracking)	7.4 ± 0.4	18.2 ± 0.7	4.3 ± 0.3	651 ± 16
−ACA (static allocation)	9.1 ± 0.4	15.6 ± 0.5	3.8 ± 0.2	635 ± 15
HPPL only (−ACA, −NDT, −WIS)	10.8 ± 0.5	18.4 ± 0.6	5.7 ± 0.3	610 ± 18

## Data Availability

Upon acceptance of this manuscript, the following artefacts will be made publicly available via a dedicated GitHub repository (HeteroEdge v1.0): (i) the W1 synthetic traffic generator (Python, configurable for all 12 protocol classes, arbitrary arrival-rate profiles, and burst injection); (ii) all testbed configuration files (DPDK ring parameters, XGBoost hyperparameter files, RTIE control parameters, WIS scenario definitions); (iii) trained XGBoost model weights and the 23-feature extraction pipeline; (iv) anonymized per-flow metadata from the W2 smart-factory trace, subject to the data-sharing agreement with the industrial partner; and (v) WIS Monte Carlo simulation scripts (SimPy). Raw packet captures from the W2 industrial trace are subject to an ongoing non-disclosure agreement and cannot be released; the anonymized metadata is sufficient to reproduce all reported classification and EPL results.

## Supplementary Materials

The following supporting information can be downloaded at: https://www.mdpi.com/article/10.3390/e28070765/s1, The Supplementary Materials contain the following figures with their complete captions: Figure S1. Workload protocol distribution with the HeteroEdge testbed topology. Distribution of network protocols in the experimental workloads deployed on the HeteroEdge testbed. The figure summarizes the protocol composition used to evaluate heterogeneous traffic processing under representative edge-network conditions. Figure S2. Annotated Anonymized Telescope Packets Sampler. Representative samples from the CAIDA Annotated Anonymized Telescope Packets Sampler dataset used for workload characterization in this study. Source: CAIDA, Annotated Anonymized Telescope Packets Sampler, accessed on 10 June 2026. Figure S3. Traffic arrival rate time series for workload W2. Temporal variation of packet arrival rates for workload W2, illustrating the dynamic traffic patterns used to evaluate adaptive strategy selection. Figure S4. Cumulative distribution function (CDF) of end-to-end parsing latency under workload W2. Comparison of the latency distributions achieved by the evaluated methods under workload W2, demonstrating differences in parsing performance across the full range of observed latencies. Figure S5. Ablation study of component contributions to latency. Impact of removing individual components of HeteroEdge on end-to-end latency, quantifying the contribution of each module to the overall performance improvements.

## Abbreviations

The following abbreviations are used in this manuscript:

ACA	Adaptive Capacity Allocation
BF	Behavioral Fingerprinting
CDF	Cumulative Distribution Function
CoAP	Constrained Application Protocol
CRDT	Conflict-Free Replicated Data Type
DES	Discrete-Event Simulation
DPI	Deep Packet Inspection
ECDF	Empirical Cumulative Distribution Function
ETSI	European Telecommunications Standards Institute
GBDT	Gradient-Boosted Decision Tree
gNB	Next-Generation Node B (5G Base Station)
gRPC	Google Remote Procedure Call
HPPL	Heterogeneous Protocol Parsing Layer
HTTP	Hypertext Transfer Protocol
IoT	Internet of Things
JA3	TLS Client Fingerprinting Method
LwM2M	Lightweight Machine-to-Machine
MEC	Multi-access Edge Computing
ML	Machine Learning
MQTT	Message Queuing Telemetry Transport
NDT	Network Digital Twin
NR	New Radio (5G Air Interface)
OPC-UA	Open Platform Communications Unified Architecture
PIM	Protocol-Independent Message
PLC	Programmable Logic Controller
QoS	Quality of Service
QUIC	Quick UDP Internet Connections
RAN	Radio Access Network
RDMA	Remote Direct Memory Access
RoCE	RDMA over Converged Ethernet
RTIE	Real-Time Inference Engine
SLA	Service Level Agreement
TCP	Transmission Control Protocol
TLS	Transport Layer Security
URLLC	Ultra-Reliable Low-Latency Communications
WIS	What-If Simulation

## Appendix A

**Table A1:** Notation used in the latency and capacity allocation model.

Symbol	Definition	Units
K	Number of protocol classes	dimensionless
M	Number of MEC nodes	dimensionless
$\lambda_{k}^{t}$	Arrival rate of protocol-k flows at time slot *t*	flows $s^{-1}$
$c_{n,k}^{t}\;$	Parsing capacity allocated to protocol *k* on node *n* at time *t*	CPU cycles $s^{-1}$
$\delta_{k}$	Mean per-packet parsing cost for protocol k	cycles ${pkt}^{-1}$
$\sigma_{k}^{2}$	Variance of per-packet service time for protocol k	$s^{2}$
$\mu_{n,k}^{t}\;$	Effective service rate $=\frac{c_{n,k}^{t}}{\;\delta_{k}}$	$pkt\;s^{-1}$
$\rho_{k}^{t}$	Per-class utilisation $=\frac{\lambda_{k}^{t}}{\mu_{\left\{n,k\right\}}^{t}}\;\in \;\left[0,1\right]$	dimensionless
$\rho^{t}$	Total $utilisation\;=\;\Sigma_{k}\rho_{k}^{t}\;\in \;\left[0,1\right]$	dimensionless
$d_{n,k}^{t}$	Mean parsing delay for protocol k at node n	s
${\bar{d}}_{n}^{t}$	Aggregate mean weighted parsing delay	s
$w_{k}$	SLA-defined priority weight for protocol k	dimensionless
$D_{\left\{n,k\right\}}^{max}$	Maximum allowable EPL for protocol k	s
$C_{n}$	Total parsing throughput budget of node n	CPU cycles $s^{-1}$
$\;c_{n,k}^{min}\;$	Anti-starvation minimum allocation for protocol k	CPU cycles $s^{-1}$
$L_{f}$	Full end-to-end latency for flow f (Equation (3))	s
$L_{radio}$	5G NR air-interface latency	s
$L_{xmit}$	Back-haul transmission latency	s
α	EWMA decay factor for NDT arrival-rate estimation	dimensionless
$J\left(c,\sigma \right)$	Expected weighted latency under strategy c in scenario σ	s
