# Supplementary material for: HeteroEdge: Latency-Aware Adaptive Protocol Parsing with Digital Twin Intelligence for Heterogeneous 5G IoT Edge Networks
Source: Entropy (Basel). 2026 Jul 3;28(7):765. doi: 10.3390/e28070765 (PMC13409465; doi:10.3390/e28070765)
Supplement: Supplementary file 1 [file entropy-28-00765-s001.zip › Figure S2.pdf]

CAIDA Acceptable Use Agreement (AUA) for Publicly Accessible Datasets

The CAIDA Acceptable Use Agreement for Publicly Accessible Datasets (Public-AUA) applies only to all publicly accessible CAIDA datasets. Other datasets are accessible under the CAIDA Master Acceptable Use Agreement (AUA). The relevant dataset agreement is shown as part of the data request process. See available CAIDA Datasets.

CAIDA ACCEPTABLE USE AGREEMENT for PUBLICLY ACCESSIBLE DATASETS

The following terms comprise the Acceptable Use Policy and Data License Agreement for all publicly accessible datasets (the "Public Agreement") made available by the Center for Applied Internet Data Analysis (CAIDA), a research unit at the University of California San Diego (UCSD) and governed by The Regents of the University of California.

LICENSE

CAIDA's authorization to access the data grants You a limited, non-exclusive, non-transferable, non-assignable, and terminable license to copy, modify, and use the data in accordance with this Public Agreement. No license is granted for any other purpose and there are no implied licenses in this Agreement. Nothing in this License is intended to limit any rights You may have arising from fair use or due to other limitations on CAIDA's exclusive rights under copyright law or other applicable laws.

CAIDA has the authority and reserves the right, in its sole discretion, to discontinue further access and use to anyone who violates this AUA.

If You create a publication (including web pages, papers published by a third party, and publicly available presentations) using data from this dataset, **You must cite the data as follows**:

> The CAIDA UCSD *[Dataset Name] - [dates used]*,
> https://catalog.caida.org/dataset/*[dataset-URL]*

You are required to report your publication to CAIDA with a copy of (or a link to) the publication. We use this information in reports to our funding agencies.

DISCLAIMER OF WARRANTIES

CAIDA USES ITS BEST EFFORTS TO PROVIDE DATA IN ACCORDANCE WITH ETHICAL PRINCIPLES AND SCIENTIFIC INTEGRITY. HOWEVER, THE DATA PROVIDED HEREIN IS ON AN "AS IS" BASIS. NEITHER CAIDA, ITS RESEARCHERS, RESEARCH PARTNERS, LICENSORS, AND DATA PROVIDERS, NOR THE UNIVERSITY OF CALIFORNIA AND ITS TRUSTEES, OFFICERS, EMPLOYEES, AND AGENTS MAKE ANY WARRANTY, EITHER IMPLIED OR EXPRESS, OF MERCHANTABILITY OR FITNESS FOR A PARTICULAR PURPOSE, INCLUDING, BUT NOT LIMITED TO, THE ACCURACY, TIMELINESS, COMPLETENESS, RELIABILITY, OR AVAILABILITY OF CAIDA DATA, APPLICATIONS, OR SERVICES ACCESSIBLE THROUGH OR MADE AVAILABLE BY CAIDA.

LIMITATION OF LIABILITY

TO THE EXTENT ALLOWED BY LAW, IN NO EVENT SHALL CAIDA AND THE UNIVERSITY OF CALIFORNIA BE LIABLE TO YOU OR ANY THIRD PARTY FOR ANY INDIRECT, CONSEQUENTIAL, INCIDENTAL, SPECIAL OR PUNITIVE DAMAGES, ARISING FROM YOUR USE OF THE DATA.

If You have any questions about the data or about this Public Agreement, please email data-info at caida.org .
